# Strong connectivity to the sensorimotor cortex predicts clinical effectiveness of thalamic deep brain stimulation in essential tremor

**DOI:** 10.1016/j.nicl.2024.103709

**Published:** 2024-11-22

**Authors:** F. Grimm, M. Walcker, L. Milosevic, G. Naros, B. Bender, D. Weiss, A. Gharabaghi

**Affiliations:** a Institute for Neuromodulation and Neurotechnology, University Hospital Tübingen (UKT), Faculty of Medicine, University Tübingen, 72076 Tübingen, Germany; b Department for Neuroradiology, University Hospital Tübingen (UKT), Faculty of Medicine, University Tübingen, 72076 Tübingen, Germany; c Center for Neurology, Department of Neurodegenerative Diseases, and Hertie Institute for Clinical Brain Research, University Tübingen, 72076 Tübingen, Germany; d Center for Bionic Intelligence Tübingen Stuttgart (BITS), 72076 Tübingen, Germany; e German Center for Mental Health (DZPG), 72076 Tübingen, Germany

**Keywords:** Essential tremor, Deep brain stimulation, Thalamus, Probabilistic diffusion tensor imaging

## Abstract

**Introduction:**

The outcome of thalamic deep brain stimulation (DBS) for essential tremor (ET) varies, probably due to the difficulty in identifying the optimal target for DBS placement. Recent approaches compared the clinical response with a connectivity-based segmentation of the target area. However, studies are contradictory by indicating the connectivity to the primary motor cortex (M1) or to the premotor/supplementary motor cortex (SMA) to be therapeutically relevant.

**Objective:**

To identify the connectivity profile that corresponds to clinical effective targeting of DBS for ET.

**Methods:**

Patient-specific probabilistic diffusion tensor imaging was performed in 20 ET patients with bilateral thalamic DBS. Following monopolar review, the stimulation response was classified for the most effective contact in each hemisphere as complete vs. incomplete upper limb tremor suppression (40 assessments). Finally, the connectivity profiles of these contacts within the cortical and cerebellar tremor network were estimated and compared between groups.

**Results:**

The active contacts that led to complete (n = 25) vs. incomplete (n = 15) tremor suppression showed significantly higher connectivity to M1 (p < 0.001), somatosensory cortex (p = 0.008), anterior lobe of the cerebellum (p = 0.026) and SMA (p = 0.05); with Cohen’s (d) effect sizes of 0.53, 0.42, 0.25 and 0.10, respectively. The clinical benefits were achieved without requiring higher stimulation intensities or causing additional side effects.

**Conclusion:**

Clinical effectiveness of DBS for ET corresponded to a distributed connectivity profile, with the connection to the sensorimotor cortex being most relevant. Long-term follow-up in larger cohorts and replication in out-of-sample data are necessary to confirm the robustness of these findings.

## Highlights

• Identifying the optimal target for DBS placement for essential tremor is challenging.
• Previous studies on clinical response revealed contradictory connectivity profiles.
• Classification in complete vs. incomplete tremor suppression may resolve ambiguity.
• Sensorimotor connectivity is of highest relevance for complete tremor suppression.
• Benefits are not related to higher stimulation intensities or more side effects.

## 1 Introduction

Essential tremor (ET) is the most common adult tremor and one of the most common neurological disorders. (Louis and McCreary, 2021, Louis, 2023) It usually progresses slowly and often leads to a significant reduction in quality of life. (Bot et al., 2023, Gerbasi et al., 2022) To date, there is no consensus on the pathogenesis of the disease. Multiple types of abnormal brain circuitry can lead to the symptoms including a central oscillator on the olivo-cerebellar-thalamic-cortico-spinal level (Llinas and Volkind, 1973). There is increasing evidence for the involvement of the cerebellum in this neurodegenerative process (Louis and Lenka, 2017). The therapy for this hyperkinetic disorder is still purely symptomatic and effective drugs are limited. Deep brain stimulation (DBS) of the ventral intermediate nucleus (VIM) and the ventrally adjacent white matter (i.e., posterior subthalamic area) have therefore been proposed as an effective therapy in the surgical treatment of drug-refractory ET (Deuschl et al., 2011).

The VIM measures approximately 4x4x6 mm (Nowinski et al., 2008) and has low intrinsic contrast with the surrounding thalamic structures on conventional structural magnetic resonance imaging (MRI). It is anatomically described as the area of the thalamus that receives afferents from the cerebellum and then projects primarily to the motor cortices (Ilinsky and Kultas-Ilinsky, 2002). Despite the existing consensus that a precise determination of the stimulation site is crucial to the clinical success of this surgical therapy option, conventional preoperative stereotactic planning continues to be based on indirect, atlas-based targeting methods that elude important inter-and intra-individual anatomical and functional differences (Akram et al., 2018, Middlebrooks et al., 2018, Anderson et al., 2011). However, an increasing number of centers are implementing novel targeting methodologies that take into account anatomical variability through the use of patient-specific imaging, such as tractography or advanced imaging sequences. (Deuschl et al., 2011, Bot et al., 2023) To date, diffusion tensor imaging (DTI) is the only non-invasive method for depicting white matter tracts in the human brain. Probabilistic tractography considers fibers crossing within a voxel (Behrens et al., 2003) to estimate the paths emanating from each voxel. It provides quantitative information on the probability of structural connectivity to a defined target region. The first in vivo connectivity-based segmentation of the thalamus by Behrens et al. (Behrens et al., 2003) formed the basis for numerous subsequent connectivity studies. Probabilistic tractography was used to analyze the connectivity profile between VIM and cortical and cerebellar structures (Hyam et al., 2012, Klein et al., 2012, Groppa et al., 2014) or to segment the thalamus based on corresponding thalamo-cortical connectivity (Akram et al., 2018, Pouratian et al., 2011, Middlebrooks et al., 2018). The basic idea for the use of DTI tractography in DBS targeting is that the anatomical network connectivity of a target region could be a more precise predictor of efficacy than its histochemical properties. The latter are used to classify thalamic nuclei in common atlases (Hirai and Jones, 1989) which, in turn, serve as the basis for preoperative stereotactic planning of target regions in DBS to this day. However, one major limitation of this atlas-based approach of thalamic segmentation is its inability to discern thalamic substructures using conventional imaging techniques.

The question as to which cortical areas in thalamo-cortical fiber tracking are most relevant for the clinical outcome of thalamic DBS is currently a subject of debate in the literature (Akram et al., 2019, Middlebrooks et al., 2019). Non-human primate studies (Sakai et al., 2000), anatomical and neurophysiological human studies (Hellriegel et al., 2012), together with one magnetoencephalography (MEG) study (Hartmann et al., 2018) emphasize the importance of connectivity to the primary motor cortex. This is supported by earlier DTI-based connectivity work on the clinically most effective stimulation site in thalamic DBS (Akram et al., 2018, Anderson et al., 2011, Hyam et al., 2012, Klein et al., 2012, Groppa et al., 2014). However, other connectivity studies postulated that the most effective thalamic area is strongly associated with the supplementary/premotor cortex (Pouratian et al., 2011, Middlebrooks et al., 2018).

In the present study, we aimed to resolve some of the ambiguities mentioned above. For this purpose, we differentiated stimulation contacts in each hemisphere by applying (unlike earlier studies) a binary classification of stimulation responses based on the clinical outcome: complete vs. incomplete tremor suppression. Specifically, we hypothesized that incomplete tremor suppression, despite optimized stimulation programming, would indicate a relevant distance to the best therapeutic spot within the tremor network. Accordingly, we conjectured a significant difference in the respective DTI connectivity profiles of active contacts between these two groups. Even though incomplete tremor suppression may also lead to patient satisfaction and improvement of quality of life, we postulate that the applied analysis approach − when identifying significantly different connectivity profiles − would contribute to the optimization of preoperative targeting in ET patients treated with thalamic DBS in the future.

## 2 Material and methods

### 2.1 Study cohort

This study was approved by the local ethics committee of the Medical Faculty of the University of Tübingen in accordance with the Declaration of Helsinki. We retrospectively analyzed 20 medically-refractory patients with ET, all of whom had undergone bilateral thalamic DBS surgery. This subgroup of thalamic DBS patients was selected based on the availability of sufficient diffusion-weighted imaging for comparisons with the clinical outcome. The decision in favor of stereotactic thalamic DBS surgery was taken by an interdisciplinary review board (neurology, neurosurgery, psychiatry, neuroradiology, anesthesiology and cardiology) of the University Hospital Tübingen.

### 2.2 Operative procedure

Trajectory planning was performed preoperatively using standard ac-pc (anterior commissure – posterior commissure) coordinates sets (5 mm anterior to pc; −14 mm lateral to the midline; at ac/pc level) and adjusted on the basis of individual anatomy based on T1-weighted 3D data. Thereby, the ventral intermediate nucleus (VIM) served as the initial target point. Stereotactic electrode implantation (Medtronic DBS Lead 3389: n = 36, Boston Scientific Vercise Cartesia: n = 4) was performed as awake surgery. To enable a direct data comparison of the stimulation amplitudes of both stimulators (Medtronic and Boston Scientific), we transformed the current values of the latter to volts on the basis of Ohm's law and the individual impedances. The impedances were measured prior to the clinical evaluation, which took place, on average, nine weeks after surgery. This measurement was completed immediately prior to the stimulator being turned off for the pre-evaluation washout. It is important to note that the current-to-voltage transformation was performed for the purpose of comparing stimulation amplitudes, rather than for the estimation of VTAs. This calculation would have limitations in accurately determining precise VTAs. Accordingly, the seed region for tractography was defined as a 1 mm sphere around the active contact in all cases.

The intraoperative control was performed by x-ray, local field potential (LFP) recordings and clinical testing with stimulation to assess tremor suppression and side effects. During surgery, the lead was moved beyond the initial target point (i.e., VIM) into the ventrally adjacent white matter. This adjustment was guided by LFP monitoring from the DBS contacts, with intraoperative power calculations (Milosevic et al., 2020, Belardinelli et al., 2020) identifying changes in background activity at the thalamus-white matter boundary, to position the lowest contact below the VIM.

### 2.3 Clinical evaluation

As per our center's standard operating procedure, patients underwent an initial contact review immediately after surgery and were discharged with the DBS active. The comprehensive monopolar review was conducted during a subsequent visit, on average nine weeks later. Prior to this review, a washout period was introduced to allow sufficient time for the effects of any previous stimulation settings to subside before further measurements or adjustments were made. At this stage, a comprehensive assessment was performed to refine the programming, including measurement of the impedances of the electrode contacts to assess their integrity. During these adjustments, stimulation programming was optimized to reduce or remove side effects while preserving or improving tremor suppression.

It should be noted that, differently than sensor-based continuous tremor assessments, ordinal scale-based rating scales (e.g., slight, moderate, marked tremor) are susceptible to subjective variability. Furthermore, there is a possibility that the examiner's evaluation and the patient's perspective may differ. To mitigate this potential bias, we applied a binary classification of the clinical outcome: complete vs. incomplete tremor suppression. This classification was performed by taking the patient's self-report and the clinical assessment of the neurological specialist into account. Complete tremor suppression was documented only when this was reported by both the patient and the examiner.

The monopolar review was conducted after stimulation was off for at least 15 min prior to the examination unless patients could not tolerate tremor rebound. The upper extremity tremor was assessed in the holding posture (arms outstretched, wrists mildly extended, fingers spread apart). All four ring levels per lead were evaluated with omnidirectional stimulation in random order with a frequency of 130 Hz and a pulse width of 60 µs, while increasing the stimulation amplitude at 0.5 mA increments with a washout period of 30 sec between evaluations. When complete tremor suppression was achieved at more than one ring level, the level achieving this effect with the lowest stimulation amplitude was defined as the best one (i.e., active contact), and amplitude titration was continued at 0.1 mA increments to identify the exact threshold that would minimize side effects while preserving complete tremor suppression. Following this optimization process, the patients had no or tolerable side effects (Table 1).

**Table 1:** Detailed demographic and clinical information of the patient sample.

Patient		ET1	ET2	ET3	ET4	ET5	ET6	ET7	ET8	ET9	ET10	ET11	ET12	ET13	ET14	ET15	ET16	ET17	ET18	ET19	ET20	Mean
**age**		62	53	73	65	51	60	79	72	82	74	78	56	71	74	78	71	77	75	71	72	70
**gender**		m	m	w	m	m	m	m	m	m	w	m	w	m	w	m	w	m	m	w	m	
**disease duration (years)**		20	8	22	50	6	2	8	5	10	7	8	44	56	24	36	5	14	15	20	15	18.75
**follow up (weeks)**		8	13	9	10	6	9	7	8	5	10	8	12	8	9	8	9	7	8	20	9	9
**clinical benefit**	left	1	1	0	1	1	1	0	0	1	1	0	1	0	1	0	0	1	1	1	1	0.65
	right	0	1	0	1	1	1	1	1	1	1	0	0	0	0	0	0	1	1	1	1	0.6
**Side effects**		dysarthria. depressive symptoms	dysarthria. gait ataxia	dystonia (mainly of the left leg)	mild dysarthria	mild dysarthria	none	mild dysarthria	none	mild dysarthria	exacerbation of a pre-existing depression	none	none	mild dysarthria	none	none	none	facial paresthesia	facial paresthesia	none	none	
**active contacts**	left	1	2	1	1	1	1	1	3	1	1	2	1	1	2	1	1	1	2	2	1	1.35
	right	1	2	1	1	1	1	1	1	2	1	1	1	1	2	1	1	1	2	2	1	1.25
	AMP (volt) left	3.6	2.8	2	1.8	3.8	1.7	2.8	3.5	2.3	3.5	2.5	2	2.2	3	2	1.6	3	3.3	2.8	1.5	2.585
	AMP (volt) right	3.6	4.4	1.8	1.8	3.3	1.7	1.4	2	3.2	5	1.6	2.1	2.2	3.4	2.5	1.6	1.5	2.9	3	1	2.5
	PW (μS) left	90	120	60	120	60	60	60	210	60	120	60	60	60	60	90	60	60	90	90	90	84
	PW (μS) right	90	120	60	120	60	90	60	120	90	120	60	60	60	40	90	60	60	90	90	90	81.5
	FREQ (Hz) left	130	130	130	130	150	140	130	130	130	90	150	130	130	130	130	130	130	130	130	150	131.5
	FREQ (Hz) right	130	130	130	130	150	140	130	130	130	90	150	130	130	130	130	130	130	130	130	150	131.5

When complete tremor suppression was not achieved at a particular electrode lead with 130 Hz and 60 µs, frequency and pulse width were increased at the ring level at which the effects on tremor were strongest (i.e., active contact). Only if tremor was still present after testing different intensities, frequencies and pulse widths at this active contact, was the lead categorized as “incomplete tremor suppression”. This approach generated a broad spectrum of stimulation parameters, encompassing voltages from 1.4 V to 5 V, frequencies ranging from 90 Hz to 150 Hz, and pulse widths between 40 μS and 120 μS, irrespective of whether full tremor suppression was achieved (Table 1). Accordingly, no significant differences in stimulation parameters were observed between the two groups (see Results). Each patient was then assigned an ordinal score per hemisphere/active contact on the basis of the clinical outcome (n = 40 scores; 2 hemispheres x 20 patients): 1 for complete (n = 25) and 0 (n = 15) for incomplete tremor suppression of the upper limb. To avoid overflow effects, one hemisphere/active contact was classified as “incomplete tremor suppression” only, if the tremor persisted despite simultaneous stimulation in the contralateral hemisphere/active contact with optimized parameters. The connectivity profiles of these active contacts (complete vs. incomplete tremor suppression) were compared to each other, assuming significant differences independent of the respective programming parameters (e.g., stimulation amplitudes).

We are aware that habituation effects may occur over time, leading to a decrease of responsiveness with longer clinical follow-up periods (Anthofer et al., 2017, Eisinger et al., 2018). Moreover, disease progression contributes to the multifactorial nature of postoperative tremor outcome. Therefore, the variability of responses to the very same stimulation is liable to increase over time, thereby blurring the impact of the lead position on clinical outcome. Furthermore, electrode insertion into the target area has been observed to induce a degree of clinical improvement even before the initiation of stimulation. (Hamani et al., 2024) Studies in ET patients indicate that this effect occurs in approximately 50 % of cases, with an average duration of 25 days. (Sitburana et al., 2010) We therefore decided to evaluate the patients several weeks after surgery, when the insertional effect had dissolved and the effects of habituation and disease progression had not yet set in.

### 2.4 Imaging

As in earlier work in this field (Strotzer et al., 2019), high-resolution MRI of the brain was performed preoperatively in a 1.5 Tesla magnetic resonance tomograph (Aera/Avanto, Siemens Healthineers, Erlangen, Germany). Our protocol includes a contrast-enhanced sagittal T1-weighted Fast Low-Angle Shot 3D (FLASH 3D) sequence (176 slices, repetition time = 1400 ms, echo time = 2.52 ms, flip angle = 15°, matrix size = 256x256, voxel size = 1x1x1mm^3^). The DWI was acquired with a single-shot spin-echo echo planar imaging (SS SE-EPI) (repetition time = 4700 ms, echo time = 79 ms, matrix size = 256x256, voxel size = 2x2x2mm^3^, 30 diffusion directions, gradient direction b = 1000 s/mm^2^).

Postoperatively, CT images (220 slices, matrix size = 512x512, resulting in a reconstruction diameter of approximately 0.43x0.43x1mm^3^) of the cranium were performed in a Somatom Definition AS + Scanner (Siemens Healthineers, Erlangen, Germany) and used for lead localization after co-registration with the preoperative MRI.

### 2.5 Data analysis

#### 2.5.1 Preparation of seed and target masks

To determine the contact coordinates, postoperative CT images were first merged with the preoperative T1-weighted imaging using the iPlan Stereotaxy 3.0 software package (BrainLAB, Feldkirchen, Germany). The midpoint coordinate of the lowest contact was identified, and the electrode vector was determined via a second point along with the electrode artifact. From these data, the center point coordinates of the other three contacts were trigonometrically calculated, taking the electrode specifications into account (1.5 mm contact length, 0.5 mm inter-contact distance). Further analyses were performed with the software package FMRIB Software Library v6.0 (FSL) (Jenkinson et al., 2012). Calculations were performed in the individual patient space (high-resolution preoperative T1-weighted data). The contact coordinates were used to mask the seed points by FSLmath with a radial sphere of 1 mm radius.

**Fig. 1:**
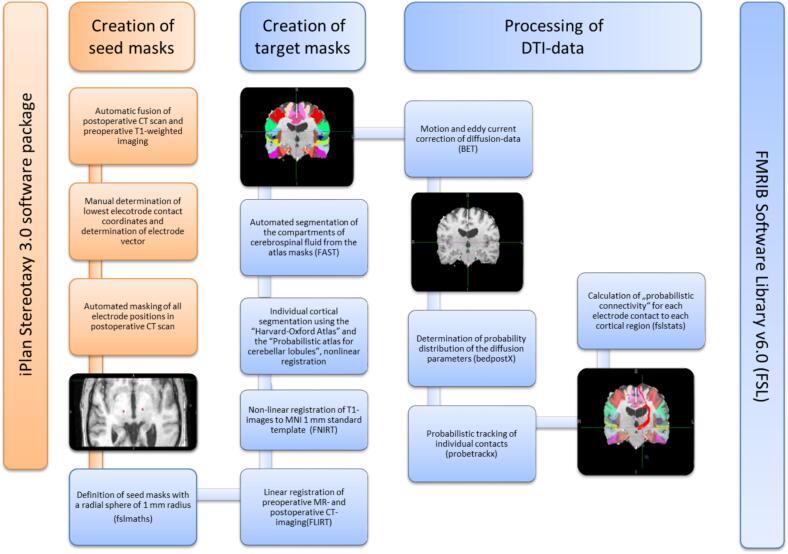
Flow chart of the processing steps.

To create the target masks, an affine transformation was first performed from MNI to native space (Jenkinson et al., 2002). This was followed by a non-parametric transformation (Andersson et al., 2010) to individual patient space of both the Harvard-Oxford cortical and subcortical structural atlas (Desikan et al., 2006) and the probabilistic atlas for cerebellar lobules (Diedrichsen et al., 2009) including the deep cerebellar nuclei (Diedrichsen et al., 2011). The compartments of cerebrospinal fluid (CSF) that had previously been determined with the FMRIB's Automated Segmentation Tool (FAST) (Zhang et al., 2001) were subtracted from the atlas masks. An overview of all processing steps is shown as a flowchart in Fig. 1.

### 2.6 Processing of DTI-data

Data were corrected for eddy current-induced distortions (Andersson and Sotiropoulos, 2016). In preparation for probabilistic tractography, the probability distribution of the diffusion parameters – and thus of the underlying fiber directions – was determined by applying the bedpostx algorithm (Bayesian Estimation of Diffusion Parameters Obtained using Sampling Techniques) which performs the Markov-Chain-MonteCarlo sampling procedure (Behrens et al., 2003, Behrens et al., 2007).

Probabilistic tractography was performed using the program probtrackx of the FMRIB Diffusion toolbox, and applying the parameters used by Behrens et al. (Behrens et al., 2007) (step length 0.5 mm, number of samples: 5000, 0.2 curvature threshold, loop-check termination, maximal number of steps: 2000, subsidiary fiber volume fraction threshold: 0,01, waypoint options: Apply waypoint independently in both directions). The spheres around the contact center defined above were selected as the initial structure (mode = seedmask).

**Fig. 2:**
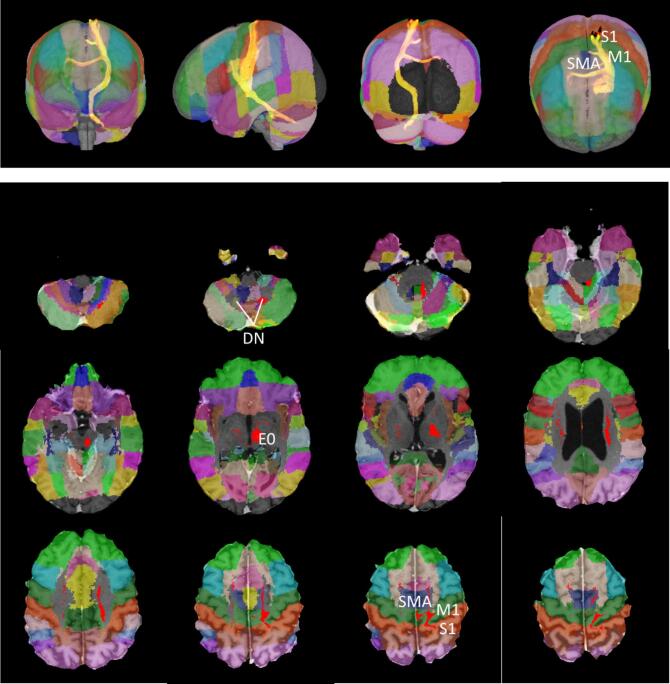
Three-dimensional display of connectivity from electrode E0 to the cortex of an exemplary patient (ET01). Upper panel: superimposed 3D representation of all probabilistic tracts originating from E0. From left to right: View from frontal, lateral, dorsal and superior. Lower panel: axial slices from caudal to cranial of the same tract in individual patient space.

The probabilities of the connectivity were calculated for each active (i.e., most effective) contact to the 48 cortical and 20 cerebellar target regions (per hemisphere) (Fig. 2). The mean value of the voxels other than zero was used as a measure of the probability of existing connectivity between the contacts and the previously defined cortical areas. This approach will henceforth be termed “probabilistic connectivity” (PC).

### 2.7 Statistical analysis

Statistical analysis was performed in SPSS (IBM SPSS Statistics for Windows, Version 22.0. Armonk, NY: IBM Corp.). The statistical evaluation of the patient data was descriptive, whereby arithmetic mean, median, minima and maxima (age, sex) were determined. A significance level of 5 % was assumed for the tests (α = 0.05).

First, the data were examined for normal distribution using the Kolmogorov-Smirnov test and the Shapiro-Wilk test. The non-normally distributed data were tested using the nonparametric Mann-Whitney-*U* test. This procedure enabled us to compare the groups (i.e., complete vs. incomplete tremor suppression) with regard to the probabilistic connectivity of their active contacts to the cortical areas M1, S1 and SMA and the cerebellum. These areas were selected on the basis of the literature pertaining to the tremor network and previous connectivity analyses in ET patients with DBS (for an overview see: (Sharifi et al., 2014, Wong et al., 2020). Furthermore, additional areas with connectivity values similar to the selected areas (Fig. 2) were considered for secondary analysis (precuneus and posterior division of the cingulate gyrus). Cohen's d classification was used to assess the magnitude of the effect (d > 0.2: small, > 0.5: medium, > 0.8: strong).

The stimulation voltage had no outliers in the data, as assessed by inspection of the boxplot. The stimulation voltage was normally distributed, as assessed by Shapiro-Wilk's test (p > 0.05). There was homogeneity of variances, as assessed by Levene's test for equality of variances (p > 0.05). Nonparametric tests were conducted to determine whether the stimulation voltage influenced side effects or the stimulation effectiveness.

A chi-square test of homogeneity was performed to ascertain whether the proportions between the stimulation effectiveness and the occurrence of side effects differed between groups.

### 2.8 Postoperative lead localization

The coordinates of the active contacts were determined with respect to the ac/pc line which was standardized to a length of 26 mm; anterior commissure (0/0/0), posterior commissure (0/-26/0). Standardization of the contact position was performed by back transforming the coordinates from native space to MNI-space. We compared the absolute lateral distance from midline, anterior-posterior position, and depth of each of the active contacts between the two groups (1: complete tremor suppression; 0: incomplete tremor suppression). The Kolmogorov-Smirnov test was used to examine each of the three datasets for normal distribution. Since all data were normally distributed, the groups were compared using 2-tailed heterostatic t-tests.

## 3 Results

The mean age of the patients at the time of surgery was 69.6 ± 8.6 years (M ± SD, mean ± standard deviation). The gender distribution was 6 (30 %) female and 14 (70 %) male patients. Table 1 (at the end of the document) provides detailed demographic and clinical information.

### 3.1 Connectivity profile

**Fig. 3:**
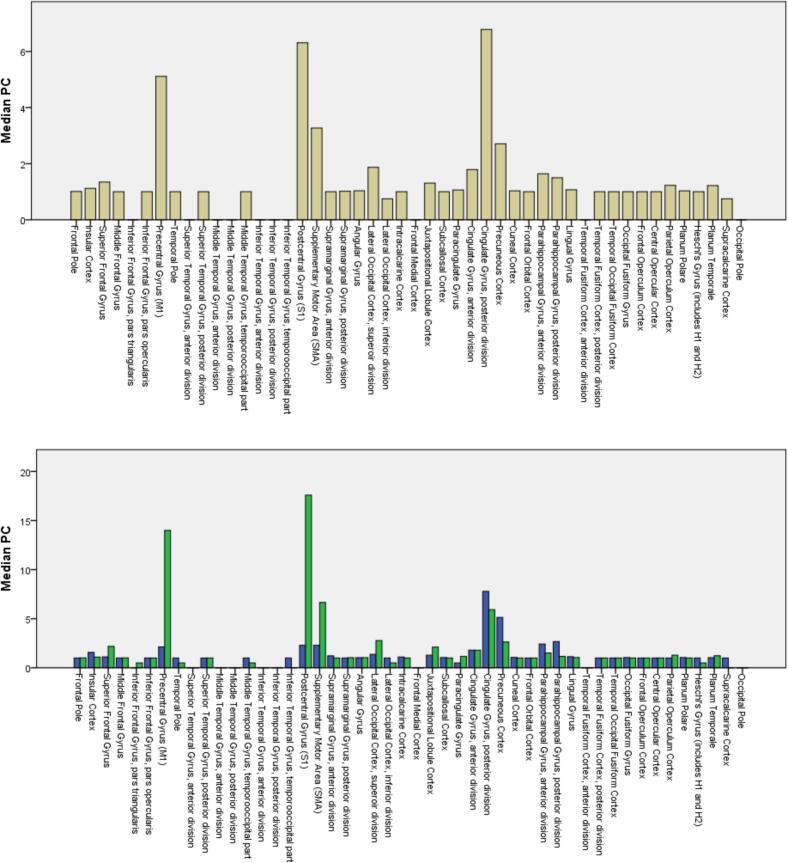
Bar chart of the median probabilistic connectivity (PC) to 48 cortical areas. Upper panel: all contacts. Lower panel: contacts with complete (green) and incomplete (blue) tremor suppression. (For interpretation of the references to color in this figure legend, the reader is referred to the web version of this article.)

**Fig. 4:**
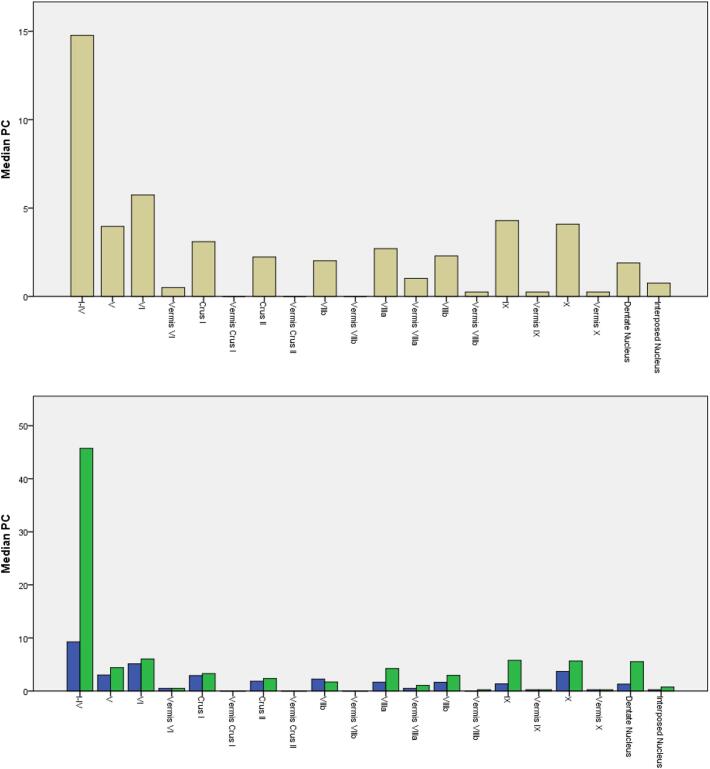
Bar chart of the median probabilistic connectivity (PC) to 20 cerebellar areas. Upper panel: all contacts. Lower panel: contacts with complete (green) and incomplete (blue) tremor suppression. (For interpretation of the references to color in this figure legend, the reader is referred to the web version of this article.)

With regard to significant differences for complete vs. incomplete tremor suppression, connections of the active contacts to the primary motor (Cohen’s (d): 0.53) and somatosensory cortex (0.42) showed the largest effect sizes, followed by the anterior lobe of the cerebellum (0.25). The supplementary motor cortex revealed significant differences for complete vs. incomplete tremor suppression also, but with only a very small effect size (0.10), whereas findings for the dentate nucleus were non-significant. A secondary analysis showed high connectivity to the precuneus cortex and posterior division of the cingular gyrus with an inverse relationship to clinical effectiveness, albeit without significant differences between complete vs. incomplete tremor suppression. The median probabilistic connectivity (PC) values of the active contacts to cortical and cerebellar areas are shown in Fig. 3, Fig. 4, respectively. The statistical comparisons are shown in Table 2. The gender distribution was 6 (30 %) female and 14 (70 %) male patients. The mean age of the patients at the time of surgery was 69.6 ± 8.6 years (M ± SD, mean ± standard deviation). Table 1 (at the end of the document) provides detailed demographic and clinical information.

**Table 2:** Group comparison of probabilistic connectivity (PC). Bold font indicates significant differences between complete and incomplete tremor suppression. “*” indicates medium and small effect sizes, with a Cohen’s (d) of > 0.5 and > 0.2, respectively.

Target area	Overall median PC	Group median PC incomplete tremor suppression	Group median PC complete tremor suppression	Exact Mann-Whitney-*U* test	Effect size Cohen’s (d)
Primary motor cortex (M1)	5.1	2.1	14	U = 308, **p < 0.001**	0.53*
Somatosensory cortex (S1)	6.3	2.2	17.6	U = 282, **p = 0.008**	0.42*
Cerebellar Lobules I-IV	14.8	9.2	45	U = 267, **p = 0.026**	0.25*
Suppl. motor area (SMA)	3.2	2.2	6.6	U = 258, **p = 0.05**	0.10
Dentate Nucleus	1.8	1.3	5.5	U = 243, p = 0.13	0.05
Precuneus cortex	2.7	5.1	2.6	U = 201, p = 0.7	0.11
Cing. Gyrus post. division	6.7	7.7	5.9	U = 198, p = 0.7	0.04

### 3.2 Lead locations

The mean lateral distances from midline were 13.5 ± 2.0 mm and 14.1 ± 2.6 mm (p = 0.4887); the mean distances to the anterior commissure were −16.9 ± 1.5 mm and −17.1 ± 2.6 mm (p = 0.6763); and the mean depths with respect to the ac/pc line were −1.81 ± 2.5 mm and 0.50 ± 2.7 mm (p = 0.1367) for the complete and incomplete tremor suppression groups, respectively (mean ± standard deviation). While the locations of the active contacts did not differ significantly between groups, a significant difference (p = 0.0286) was detected for the overall electrode depth; i.e., the average positions of the ventral-most contact with respect to the ac/pc line were −3.12 ± 2.1 mm and −1.17 ± 2.8 mm for complete and incomplete tremor suppression, respectively.

**Fig. 5:**
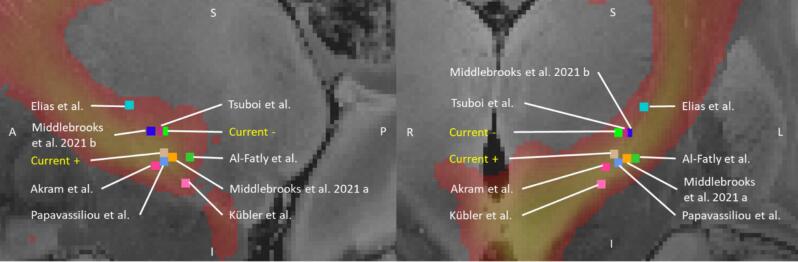
The contact locations in the current study with complete (current + ) and incomplete tremor suppression (current −) are shown in comparison to sweetspots as reported in previous studies (Elias et al. (Elias et al., 2021), Tsuboi et al. (Tsuboi et al., 2021), Al-Fatly et al. (Al-Fatly et al., 2019), Akram et al. (Akram et al., 2019), Papavassiliou et al. (Papavassiliou et al., 2004), Kübler et al. (Kubler et al., 2021), Middlebrooks et al. 2021 a (Middlebrooks et al., 2022) and b (Middlebrooks et al., 2021),and summarized by Middelbrooks et al. 2021b (Middlebrooks et al., 2021). The DRT pathway, as derived from Dembek et al., (Dembek et al., 2020) is depicted in color coded form in both sagittal (left) and coronal (right) orientations. The background is the normalized FLASH25 dataset (Edlow et al., 2019).

A comparison of the mean coordinates of the complete and incomplete tremor suppression groups with the coordinates reported in previous studies for optimal response (“sweet spots”) (Akram et al., 2019, Elias et al., 2021, Tsuboi et al., 2021, Al-Fatly et al., 2019, Papavassiliou et al., 2004, Kubler et al., 2021, Middlebrooks et al., 2022, Middlebrooks et al., 2021) and the spatial relationship with the dentatorubrothalamic (DRT) pathway (Dembek et al., 2020) are shown in Fig. 5. The coordinates of both groups in this study are situated in close proximity to the previously reported sweet spots and overlap with the DRT. This suggests that the cortical connectivity profile identified in this study provides additional information for differentiating between complete and incomplete tremor suppression, which is not sufficiently covered by the proximity to the DRT alone.

### 3.3 Stimulation voltage, clinical effectiveness and side effects

The mean stimulation voltage in patients with side effects (2.82 ± 0.95 V) was 0.63 ± 0.27 V higher than for side effect-free stimulation (2.19 ± 0.71 V). This difference was statistically significant (t (38) = 2.33, p = 0.025). However, the proportion of patients with side effects did not differ significantly between the groups with complete and incomplete tremor suppression (p = 0.125).

The mean stimulation voltage for the group with complete tremor suppression (2.65 ± 1.0 V) was 0.29 ± 0.29 V higher than for the group with incomplete tremor suppression (2.36 ± 0.69 V). There was, however, no statistically significant difference between groups (t (38) = 0.99, p = 0.328).

## 4 Discussion

In this study, probabilistic DTI-based tractography was used to establish patient-specific connectivity profiles that were indicative of complete (vs. incomplete) tremor suppression in 20 bilateral thalamic DBS patients with ET. The active contacts that led to complete vs. incomplete tremor suppression showed significantly higher connectivity to M1, somatosensory cortex, anterior lobe of the cerebellum and SMA; however, the different effect sizes suggest that the sensorimotor connectivity is of highest relevance. The stimulation voltage was significantly higher in patients with side effects. However, the stimulation voltage was not significantly different between groups. Furthermore, the proportion of patients with side effects did not differ between groups. This suggests that complete tremor suppression was not achieved at the expense of side effects, thereby indicating a clinically effective DBS connectivity pattern. The active contacts that led to complete tremor suppression were numerically but non-significantly more medial, anterior and inferior to those with incomplete suppression. Notably, the respective leads were located significantly deeper, an observation that requires further investigation in the context of identifying the anatomical substrates of tremor suppression in the (sub)thalamic area (Middlebrooks et al., 2019, Al-Fatly et al., 2019).

Previous studies in this field used a tremor rating scale such as that of Fahn, Tolosa and Marin (Fahn et al., 1993) to assess the DBS response. However, these subjective evaluations (e.g., estimating slight, moderate, marked tremor) are – other than objective and continuous sensor-based measures − affected by variability and suggest a precision that they cannot provide. Albeit relevant for the quantification of clinical outcome, rating scales may thus introduce additional complexity to the evaluation of connectivity-structure relationships and between-study comparisons, since different studies base their correlation analysis on different clinical improvements. Specifically, some studies report tremor reduction following thalamic DBS of 34 %, 41 %, or 63 % on the group level, which may be partly related to different follow-up time points following surgery (Akram et al., 2018, Pouratian et al., 2011, Middlebrooks et al., 2018, Al-Fatly et al., 2019). However, this variability of response rates indicates that relative clinical effectiveness, e.g., 40 % tremor reduction, in one study may be considered relatively ineffective in another. The respective correlation analysis with DTI connectivity may therefore also be biased. Specifically, the connectivity profile of, e.g., 40 % tremor reduction, may represent the “upper border” in one study, i.e., being considered as effective connectivity, while representing the “lower boarder” in another study, i.e., being considered as ineffective connectivity. In the present work, we thus applied a binary classification (complete vs. incomplete tremor suppression) which is rather unambiguous and also easily applicable to already existing datasets; this will allow for direct comparisons across studies in future.

In recent years, numerous DTI-based connectivity studies have been conducted in the context of thalamic DBS. These were based on the pathophysiological concept that a cerebello-thalamo-cortical tremor network plays a crucial role in mediating abnormal oscillatory tremor activity; along these lines, modulation of this network – indexed by a strong connectivity between active DBS contacts and network nodes – is expected to determine the therapeutic effects of DBS (Al-Fatly et al., 2019). While some approaches investigated the connectivity between specific nodes of this network (Anthofer et al., 2017, Nowacki et al., 2018), others examined whole-brain connectivity patterns on the basis of patient-specific (Akram et al., 2018, Pouratian et al., 2011, Middlebrooks et al., 2018) or normative connectome data (Al-Fatly et al., 2019). Ambiguities between studies may be related to methodological differences with regard to the investigated data and respective data processing approaches.

### 4.1 Data processing

With regard to data processing, the different approaches for determining seed regions make it difficult to draw comparisons between the studies. In many studies, a “volume of tissue activated (VTA)” is modeled on the basis of the stimulation parameters and the electrical properties of the surrounding structures. This entails the application of different algorithms, which may thus lead to an over- or underestimation of the actual seed region (Horn et al., 2017, McIntyre and Foutz, 2013, Astrom et al., 2015). In the present work, the seed region was defined as a sphere around the active contact, thereby simplifying the actual activation pattern. However, the method of probabilistic DTI considers neighboring voxels of its own accord. If the seed region were to be additionally enlarged according to a VTA, its boundary areas would increase exponentially. This effect is amplified by the somewhat coarse voxel resolution of DTI that would result in further overrepresentation and distortion of the seed volume and increase the uncertainty of the analysis. Moreover, the average coordinates of the two groups were in close proximity, with mean distances of 0.6 mm, 0.2 mm, and 2.3 mm in the x-, y-, and z-plane, respectively. Since the stimulation was conducted with 2.65 ± 1.0 V and 2.36 ± 0.69 V, this would result in a significant overlap of the VTAs and limit the specificity with which the connectivity profiles could be established. This limitation was addressed by implementing a 1 mm sphere around the active contacts, which enabled a more precise connectivity analysis. Furthermore, tractography has inherent limitations with regard to the medial–lateral axis of the defined cortical target region, leading to erroneous representation of cortical areas in the thalamus (Akram et al., 2019).

These aforegoing studies have therefore led to different results in determining the most favorable areas for a good clinical outcome after DBS. On the one hand, this variability highlights the complexity of targeting within and around the thalamus for ET (Wong et al., 2020). On the other hand, some of the studies used a variety of technological (data of variable spatial and angular resolution) and methodological approaches (volumes of tissue activated, stimulation settings, clinical follow-up periods and outcome variables) that may have contributed to the mixed findings (Wong et al., 2020). Therefore, while we appreciate that different stimulation parameters will result in different tissue propagation of the electrical stimulation, we suggest that our conservative approach of using a fixed volume combined with a binary classification based on clinical outcome facilitates the usability of our approach. However, we recognize that estimating the volume of activated tissue or assessing differential pathway activation, especially in the case of directional stimulation, could provide valuable insights beyond those offered by our current approach. (Gharabaghi et al., 2024) In the majority of earlier studies, the primary motor cortex was identified as the cortical area with the highest connectivity to clinically effective contacts (Anderson et al., 2011, Klein et al., 2012, Groppa et al., 2014). This can be reconciled with anatomical knowledge (Morel et al., 1997), data from non-human primate studies (Sakai et al., 2000, Strick, 1976), as well as from numerous anatomical and neurophysiological studies including MEG analysis (Hellriegel et al., 2012, Hartmann et al., 2018, Raethjen and Deuschl, 2012).

### 4.2 *Normative* vs*. patient-specific data*

The functional connectivity maps based on normative DTI data (Al-Fatly et al., 2019) demonstrated that multiple regions (M1 and S1, visual cortices V1 and V2, superior temporal gyrus, superior and inferior cerebellar lobules and, to some extent, the premotor cortex and SMA) are associated with the active DBS contacts. Although these results are in line with our findings with regard to the importance of connectivity with M1, S1, cerebellum, and to a lesser extent with SMA, we did not identify any notable connectivity with visual cortical areas or superior temporal gyri. Their structural connectivity profile further highlighted the superior parietal lobule (not explicitly investigated in our study) and precuneus (corroborated by our findings, albeit non-significant). The normative connectome data (Al-Fatly et al., 2019) also demonstrated a positive correlation between their overall multi-site functional connectivity profile and clinical outcome. In contrast to our study which relies on a seed-ROI analysis, they used a voxel-wise analysis based on linear regression to scale the importance of the voxels regarding clinical effectiveness.

Patient-specific DTI data (Akram et al., 2018) demonstrated that probabilistic tractography can be used to segment the thalamus on the basis of cortical and cerebellar connectivity. This work demonstrated distinct M1, S1 and SMA/premotor-related thalamic segments. Furthermore, fibers of the contralateral dentate nucleus were shown to first pass through the ipsilateral red nucleus, and then the thalamic region representing VIM (overlapping with a portion of the area with M1 connectivity) before terminating in the ipsilateral M1. Specifically, the posthoc analysis of active DBS contacts revealed that good therapeutic benefit was achieved when the volume of tissue activated was within this segmented VIM area with the greatest cerebellar and M1 connectivity, whereas patients with active contacts outside of this area did not receive good clinical benefit. Corroborating these findings, an intraoperative microstimulation study (Milosevic et al., 2018) demonstrated that the most ventroposterior stimulation sites within the VIM had the greatest tremor- suppressing effects. This region of VIM, close to the ventral caudal (VC) border, corresponds to the areas with the greatest connectivity to M1 and the cerebellar dentate nucleus, and perhaps secondarily with S1 (Akram et al., 2018).

Contrary to the above findings, a further study based on patient-specific DTI (Middlebrooks et al., 2018) demonstrated that the SMA/premotor thalamic-related VTA had a significant positive correlation with tremor improvement, whereas the M1-related VTA did not. However, while the majority of patients in this study lacked significant connectivity with M1, all but one patient lacked significant connectivity with SMA/premotor cortex. It is therefore conceivable that the correlation analysis was underpowered or skewed in its ability to demonstrate the significance of M1 connectivity. Although the authors did not explicitly examine the contribution of cerebellar connectivity, they did suggest that the location of the more optimal SMA/premotor thalamic segment probably corresponded to the ventral oral anterior/posterior nuclei, which are the thalamic substructures that tend to receive more pallidal than cerebellar afferents (Ilinsky and Kultas-Ilinsky, 2002, Rouiller et al., 1994, Kuramoto et al., 2011). Contradictory as they may be, these findings are nonetheless interesting and highlight the potential role of the basal-ganglia-thalamo-cortical network in tremor suppression. Two other small cohort connectivity studies (Pouratian et al., 2011, Middlebrooks et al., 2018, Kim et al., 2018) also obtained good clinical results by stimulation of the thalamic region corresponding with the highest probability of connectivity with SMA/premotor cortex. In our study, connectivity to SMA was indeed visible in all patients, but was secondary to M1 and S1. A group comparison showed that SMA connectivity was higher in patients with a better response, comparable to the aforementioned studies. It is, however, noteworthy that connectivity to the entire sensorimotor network, including primarily M1 and S1, was accentuated in patients with complete tremor suppression. These compounds could be explained by projections over dorsal thalamic portions (Behrens et al., 2007).

### 4.3 Secondary analysis

The remaining connectivity findings in our study, which were calculated as prespecified secondary outcomes, were not included in the group comparison. Since these may be due to multiple testing, they should not be overestimated. Remarkably, however, one obvious exception was the increased overall connectivity to the precuneus and the limbic system, i.e., to the cingulate gyrus and, to a lesser extent, to the parahippocampal gyrus. Patients with incomplete tremor response showed a slightly increased connectivity to these limbic structures, although this was without statistical significance. This connectivity pattern was not investigated in previous studies with patient-specific DTI data. The projections to the limbic system are via adjacent structures such as the midline nuclei and the central medial nucleus, both of which project directly to limbic cortical structures, in particular to the hippocampus and the medial prefrontal cortex (Vertes et al., 2015). The thalamus was frequently regarded as having merely a gateway function to integrate signals to higher cortical regions. Limbic-associated regions were therefore underestimated. In the recent discourse, however, an increasing signal integration of the thalamus is described including information from the limbic system.

Both the precuneus and the posterior cingulated cortex form part of the default mode network (DMN) and play a key role in fundamental cognitive function (Cavanna and Trimble, 2006, Leech and Sharp, 2014). A voxel-wise *meta*-analysis of gray matter abnormalities in patients with essential tremor recently showed structural damage in the left precuneus extending to the left posterior cingulate gyrus (Han et al., 2018). The involvement of the precuneus in ET had already been proposed in two studies in which F-18-FDG-PET was used to show a decrease in glucose utilization in the precuneus compared to healthy controls (Ha et al., 1987, Song et al., 2015). Using functional MRI, previous studies in ET patients with low cognitive scores showed an increase in connectivity in the DMN (Passamonti et al., 2011, Benito-Leon et al., 2015). The alterations observed in the precuneus extending to the posterior cingulate cortex in the above-described studies may be linked to cognitive impairment and depressive symptoms in ET patients, both of which are common non-motor disturbances of ET (Benito-Leon et al., 2006, Benito-Leon et al., 2006, Collins et al., 2017, Louis, 2016, Louis et al., 2007, Sengul et al., 2015).

### 4.4 Cerebello-thalamo-cortical network

On a network level, the cerebello-thalamo-cortical network is closely linked to the generation of tremor, whereby the DRT pathway originates in the cerebellum (dentate nucleus) and travels to the contralateral thalamus via the red nucleus (Mollink et al., 2016, Gallay et al., 2008, Kwon et al., 2011, Brinda et al., 2023). Diffusion tensor tractography methods have elucidated that the classical targets for DBS in ET lie alongside the same cerebello-thalamo-cortical network or the DRT. Some authors therefore suggest that DRT is an effective targeting structure in the surgical treatment of ET (Coenen et al., 2014, Sajonz et al., 2016, Nowacki et al., 2022), but see also (Nowacki et al., 2018). In recent years, there has been growing evidence that ET is linked to dysfunction and a probable degeneration of the cerebellar system (Louis, 2018). Clinical and neuroimaging literature suggests that the cerebellum itself may be instrumental in the generation of ET (Sharifi et al., 2014, Benito-Leon and Labiano-Fontcuberta, 2016, Louis, 2014, Marin-Lahoz and Gironell, 2016, Filip et al., 2016, Lenka et al., 2017). In tandem with the clinical and neuroimaging studies, postmortem literature increasingly reports pathological changes in the cerebellum of patients with ET, including an increase in torpedoes (Purkinje cell (PC) axonal swellings), associated PC axonal pathologies or an increase in heterotopic PCs (Louis et al., 2007, Shill et al., 2016, Shill et al., 2008, Yu et al., 2012, Babij et al., 2013, Kuo et al., 2011). These reports reinforce the theory that the cerebellum plays a crucial role in the pathophysiology of ET, and that ET is a structural, degenerative brain disorder of cerebellar disinhibition. Such findings are in line with our results which indicate that clinically effective contacts show higher PC with cerebellar structures. With our approach, however, we showed in particular an ipsilateral increase in connectivity to the anterior lobe (lobules I-IV) of the cerebellum. While the DRT has been classically described as a decussating pathway, recent studies using deterministic fiber tractography and microsurgical postmortem dissection of human brains show that an ipsilateral or non-decussating connection also exists between deep cerebellar structures, the red nucleus and the thalamus (Meola et al., 2016, Petersen et al., 2018). However, the precise functional role of the non-decussating DRT remains elusive. The anterior lobe of the cerebellum is part of the spinocerebellum which is also connected by the emboliformis nucleus with efferents via the upper cerebellar part to the red nucleus of the opposite side. The high connectivity in the present study should therefore be regarded as a general increase in connectivity to the cerebellum since the pedunculus cerebelli is immediately adjacent and is included by the probabilistic method. The dentatorubrothalamic pathway can probably not be detected directly by the method applied in this study. In earlier work, Akram and colleagues (Akram et al., 2018) identified this tract by determining landmarks from the contralateral dentate nucleus via the complete ipsilateral thalamus to the motor cortex. The connection points of the thalamus were generally very deep, i.e., partly below the AC/PC line. These were therefore usually located below the contacts and then covered by a field modulation of the stimulated contacts in their periphery only.

If the study results are to be compared to previous work, one should first consider the methodologically different approaches of the individual studies, in particular against the background that there are no controlled-randomized studies on this topic to date. The literature on probabilistic fiber tracking in DBS shows that there is very high variability in the acquisition of imaging, partly due to the retrospective character of the studies. In the present study, the spatial and angular resolution of the underlying imaging (30 directions, layer thickness 2 mm, b-value of 1000 s/mm^2^) is relatively low compared to other studies on connectivity-based targeting in DBS (e.g., (Akram et al., 2019); 128 directions, layer thickness 1.5 mm, b-value 1500 sec/mm^3^), but comparable to others (e.g., (Pouratian et al., 2011); 20 directions, layer thickness 2 mm, b-value 1000 sec/mm^3^). In general, higher field strength and a greater number of diffusion directions lead to a higher degree of precision in the tracking results and a reduction in the scattering range of the results. This could also explain why connectivity to contralateral cerebellar structures is not apparent in the present study. While incorporating waypoint masks could help by guiding the tractography toward specific regions, this step would compromise the unconstrained approach of this study to statistically compare the probabilistic projections to all cerebral and cerebellar regions. Furthermore, it is well known that image registration steps are susceptible to geometric distortions, which can influence the spatial localization. To address this issue, all acquisitions can be repeated with reverse-phase coding to correct for possible distortions; precision may also be increased by applying multiple validations of a target area, e.g., by overlapping the M1-thalamic segment with the cerebellar-thalamic segment (Akram et al., 2019).

### 4.5 Limitations and perspectives

In general, the variation of the surgical methodology between studies, and the fact that the final electrode placement is often determined intraoperatively by electrophysiological recordings (Milosevic et al., 2018, Lenz et al., 1988) leads to a rather homogenous positional bias and limits the comparability of individual target regions. Also, in future work, patients should be studied with unilateral stimulation to avoid a possible impact of the effect of DBS on ipsilateral tremor and a potential violation of statistical assumptions of side-specific measures in patients with bilateral DBS. Furthermore, it is apparent that a low number of patients is associated with low power, which can lead to 2nd type errors. Moreover, future studies will require a systematic evaluation of longer follow-up periods at defined time points after DBS surgery to explore the long-term robustness of tremor suppression in relation to the connectivity profile (Anthofer et al., 2017, Eisinger et al., 2018). Also, DBS studies in ET that cross-validate their findings with out-of-sample data are limited, but necessary (Al-Fatly et al., 2019). Despite the above-mentioned advantages of a binary symptom classification (complete vs. incomplete tremor suppression) this approach has also limitations as it is based on subjective assessments which could be overcome by sensor-based measurements. Furthermore, it would be interesting to include a comparison of connectivity profiles and lead locations of patients with proximal vs. distal tremor, since proximal tremors are often refractory to VIM DBS. Moreover, incorporating high-field MRI, such as 3 T or 7 T, could enhance the tractography component by providing increased resolution and detail in structural connectivity. Finally, combining demographic, patient-reported, neuroimaging, and neurophysiological data to explore the response variability, along with using explainable machine learning to analyze these multimodal factors, may yield insights and predictive capabilities unattainable through any single modality alone. (Ferrea et al., 2024).

## 5 Conclusion

Complete tremor suppression following thalamic DBS corresponded to a distributed connectivity profile with graded relevance of the nodes within the tremor network; the connection between the active contact and the sensorimotor cortex was most relevant. Long-term follow-up in larger cohorts and replication in out-of-sample data are necessary to confirm the robustness of these findings.

## 6 Funding Statement

This investigator-initiated trial was supported by the European Union’s Joint Programme for Neurodegenerative Disease Research (EU-JPND 2022–130) grant Recast (01ED2309). The funding had no impact on the study design, on the collection, analysis and interpretation of data, on the writing of the report or on the decision to submit the article for publication.

## CRediT authorship contribution statement

**F. Grimm:** Writing – original draft, Methodology, Formal analysis, Conceptualization. **M. Walcker:** Writing – original draft, Formal analysis, Data curation. **L. Milosevic:** Writing – review & editing, Data curation. **G. Naros:** Writing – review & editing, Data curation. **B. Bender:** Writing – review & editing, Data curation. **D. Weiss:** Writing – review & editing, Data curation. **A. Gharabaghi:** Writing – original draft, Supervision, Project administration, Funding acquisition, Conceptualization.

## Declaration of Competing Interest

The authors declare the following financial interests/personal relationships which may be considered as potential competing interests: [A.G. was supported by research grants from Medtronic, Abbott, Boston Scientific, all of which were unrelated to this work. D. W. was supported by travel grants, speaker honoraria and research grants from Abbott, Abbvie, Bial, Boston Scientific, Medtronic, Kyowa Kirin, Stadapharm, all of which were unrelated to this work.].

## Data Availability

The data that support the findings of this study are available for researchers from the first author upon reasonable request after a formal data sharing agreement.
